# An mHealth App–Based Social Capital Intervention (PrEP US NoW) to Improve Sexual Health and Uptake of Pre-Exposure Prophylaxis Among Young, Black, Sexual Minority Men: Protocol for Intervention Development and a Pilot Randomized Controlled Trial

**DOI:** 10.2196/66326

**Published:** 2025-09-18

**Authors:** Maira Sohail, Sophia A Hussen, Sarah Dougherty Sheff, Michael Mugavero, John Schneider, Lisa Hightow-Weidman, Janet M Turan, Madeline Lynam, Latesha Elopre

**Affiliations:** 1 Department of Medicine University of Alabama at Birmingham Birmingham, AL United States; 2 Rollins School of Public Health Emory University Atlanta, GA United States; 3 Department of Medicine and Public Health Sciences The University of Chicago Chicago, IL United States; 4 College of Nursing Florida State University Tallahassee, FL United States; 5 Department of Health Policy and Organization University of Alabama at Birmingham Birmingham, AL United States

**Keywords:** young, Black, sexual minority men, the South, pre-exposure prophylaxis

## Abstract

**Background:**

Black Americans are disproportionately impacted by HIV. This disparity is more profound in the Southern United States, with the highest rates being among young, Black, sexual minority men, who are also less likely to receive state-of-the-art interventions such as pre-exposure prophylaxis (PrEP). Individual-level interventions to increase PrEP uptake do not often capitalize on the opportunity to leverage the significant effects of this group’s social networks, including Black women, on attitudes, beliefs, and behaviors around HIV prevention.

**Objective:**

To increase PrEP use, an intervention, PrEP US NoW, was designed to engage young, Black, sexual minority men’s social networks in discussions with supportive Black female facilitators and ultimately enhance their social capital.

**Methods:**

First, qualitative information on core health-promoting elements of social capital bonds was captured among young, Black, sexual minority men and Black women in extant social support networks. This information was then applied to adapt an existing, evidence-based mobile health app to create the PrEP US NoW pilot through an unblinded randomized controlled trial. Six social network groups (5 young, Black, sexual minority men + 1 Black woman) will participate in the intervention arm. These will be recruited through a network-based approach and will undergo tailored training (mobile-based and face-to-face) for app usage. At baseline, men will undergo HIV testing and both men and women will complete a sociodemographic survey. The groups in the intervention arm will engage in four 60-minute discussions led by Black women through the modified mobile health app. After the intervention, young, Black, sexual minority men will complete surveys electronically at 1 and 3 months (accompanied by HIV testing) on additional factors such as experiences of discrimination and PrEP stigma. The Black women will complete an electronic survey at 1 month, measuring feasibility and acceptability, and will participate in web-based qualitative interviews at 3 months to gain more knowledge on the PrEP US NoW facilitation process. Participants in the control arm will not engage in Black women–facilitated group discussions and will use a control version of the app. The baseline and follow-up surveys and HIV testing will be documented similarly to the intervention arm.

**Results:**

Phase 1 (development) of PrEP US NoW research activities lasted from November 2019 to June 2024. Data collection for the phase 2 randomized controlled trial began in August 2024 and is expected to be completed in December 2025. The findings will capture the intervention’s feasibility and acceptability and changes in PrEP uptake among young, Black, sexual minority men.

**Conclusions:**

The development and pilot implementation trial of the PrEP US NoW intervention is thought to leverage essential social capital among young, Black, sexual minority men, which may promote engagement in PrEP care, thus decreasing the overall number of HIV diagnoses.

**Trial Registration:**

ClinicalTrials.gov NCT07024745; https://clinicaltrials.gov/study/NCT07024745

**International Registered Report Identifier (IRRID):**

DERR1-10.2196/66326

## Introduction

Black Americans, who constitute 14% of the total US population, account for 37% of new HIV diagnoses [1]. Among Black Americans, sexual minority men account for 35% of the total new HIV diagnoses nationally, ranking as the second largest impacted group after Hispanic or Latino sexual minority men [2]. These racial disparities are more severe in the Southern United States, where nearly half of new HIV diagnoses occur [3]. In Alabama, Black Americans constitute only 27% of the total population but account for 70% of the new HIV diagnoses, with young (ages 15-29 years) Black, sexual minority men being most heavily impacted [4]. Further, Jefferson County in Alabama (which includes the Birmingham-Hoover metropolitan service areas) accounts for the highest number of new HIV diagnoses in the state, and over half of the new cases occur in young, Black, sexual minority men [5].

US federal agencies are working toward the Ending the HIV Epidemic (EHE) initiative by focusing on geographic hotspots in addition to populations that face HIV disparities [6]. The EHE initiative targets disparate populations, like young, Black, sexual minority men, in promoting the use of HIV prevention tools such as pre-exposure prophylaxis (PrEP) to reduce the number of HIV diagnoses [7-10]. Where a higher PrEP-to-need ratio indicates better PrEP coverage for those in need, recent data have suggested that Black people have the lowest PrEP-to-need ratio in the United States, 8 times lower as compared with White people [11]. These racial disparities are often a result of disparities in various crucial components of the social construct, such as racial discrimination and marginalization or homonegativity in health care settings, removing a relationship of trust, thus discouraging this population from seeking medical attention for important preventive measures, such as PrEP [12]. Recent data have suggested that people residing in the Southern United States and those aged 13-24 years are among the groups with the lowest PrEP-to-need ratio [11]. Where higher PrEP uptake has shown to reduce HIV diagnoses, poor engagement in PrEP care remains an issue, which is even more profound among young, Black, sexual minority men living in the Deep South [13,14]. This highlights the critical need for increasing PrEP engagement to decrease new HIV diagnoses among this population.

PrEP engagement can be described in terms of discrete stages constituting a PrEP Care Continuum, which includes gaining PrEP awareness, PrEP uptake (linkage to PrEP and PrEP prescription), and PrEP retention (PrEP adherence and PrEP persistence) [15,16]. Previous studies among young, Black, sexual minority men have shown that low perceived risk of HIV acquisition, poor education on sexual health, lack of information on accessing local PrEP services, concerns around PrEP-related costs or insurance, and stigma related to HIV and sexuality act as barriers to PrEP awareness and uptake [17-20]. Additionally, in populations where PrEP uptake is high, barriers to PrEP persistence remain a challenge, including stigma, low perceived HIV risk, PrEP-related side effects, PrEP-related cost, difficulty with adherence to daily medication, and attending regular PrEP-related medical visits requiring routine adjustments and availability of reliable transportation [21-23]. These persisting barriers highlight the need for additional interventions to improve PrEP care engagement.

Much work has been done around targeting social network–level factors in improving PrEP engagement [24,25]. The disproportionate burden of HIV on the young, Black, sexual minority men community in the South has been connected to intersectional stigma, a concept that elucidates how multiple stigmas occurring simultaneously worsen marginalization [26,27]. One social network-level factor that is gaining increased attention is social capital, which refers to “the sum of an individual’s resource-containing, reciprocal, and trustworthy social network connections” [28]. The social capital derived from a social network can be directly influenced by a multitude of factors, including the size and diversity of the social network, as well as knowledge and material resources owned by network members [29,30]. In other words, social networks yield social capital by providing informational, emotional, social, and instrumental support, and are thought to have beneficial effects on health behaviors, such as PrEP use [31].

Existing research has shown that Black women within young, Black, sexual minority men's social networks have the potential to enhance PrEP uptake and adherence [32,33]. For example, a randomized controlled trial conducted in Chicago leveraged young, Black, sexual minority men’s social networks to improve sexual health engagement and help with PrEP uptake [34]. This study found that PrEP use among young sexual minority men was strongly associated with network-level factors [34]. Specifically, this work highlighted the impact of ties within a social network to help with introducing new ideas, such as information on PrEP [34]. A detailed network analysis of the Chicago study also discovered that Black women were identified as the primary support figures for over half of young, Black, sexual minority men participants [34]. Our prior work exploring social networks that supported and facilitated acceptance of sexual identity among young, Black, sexual minority men participants found that both young, Black, sexual minority men and Black women functioned as main support systems [35]. Another qualitative study exploring young, Black, sexual minority men’s social networks and social capital found Black women, such as mothers and female friends, to play a major role in young, Black, sexual minority men’s social capital [36]. Together, these findings suggest the importance of Black women in providing a unique, underused health-promoting social capital for young, Black, sexual minority men. Although social network–level PrEP interventions have shown promising findings, these interventions for PrEP have been relatively underused, especially in the Southern United States.

Boosted with the fast adoption of smartphones featuring various networking apps, social networks have rapidly advanced in their ability to communicate quickly and be more interactive. While providing an excellent medium for networking, mobile apps have also shown to be an acceptable and effective method for reaching youth, including young, Black, sexual minority men, to participate in HIV testing and other HIV prevention strategies [37,38]. This suggests that mobile apps can be a useful platform for improving PrEP use among young, Black, sexual minority men and, potentially, their social networks.

Keeping in mind (1) the disproportionate impact of HIV on young, Black, sexual minority men with simultaneous underuse of PrEP in this population, (2) evidence that social networks yielding social capital can be helpful in improving PrEP use among young, Black, sexual minority men, (3) evidence that Black women play a vital role within young, Black, sexual minority men’s social networks, and (4) the success of mobile apps in improving the uptake of HIV prevention strategies, an intervention, PrEP US NoW (Pre-Exposure Prophylaxis Utilization Through Increasing Social Capital Among Young, Black, sexual minority Men Networks With Women), was designed. This intervention aims to improve PrEP uptake among young, Black, sexual minority men by engaging Black women facilitators to conduct semistructured conversations with groups of young, Black, sexual minority men around HIV prevention, sexual health, intersectional stigma, and PrEP access using a mobile health (mHealth) platform. Since the aim is to assess if social network discussions conducted within the mHealth platform would improve the social capital, beyond the existing app features, such as in-app messaging, which may also contribute, we did not add any content-based approaches to the app. The goal of this intervention is to leverage heterogeneous bonds in diverse social networks between young, Black, sexual minority men and Black women to address intersectional stigma and enhance social capital toward PrEP use.

This paper describes the PrEP US NoW protocol. Using qualitative methods, core health-promoting elements of social capital bonds between young, Black, sexual minority men and Black women in existing social networks were evaluated. This information was then applied to adapt an existing, evidence-based mHealth app to create PrEP US NoW. The intervention, piloted as a randomized controlled trial, is currently ongoing, in which the feasibility and acceptability of PrEP US NoW will be evaluated along with preliminary efficacy to increase PrEP uptake among young, Black, sexual minority men.

## Methods

### Conceptual Framework

**Figure 1:**
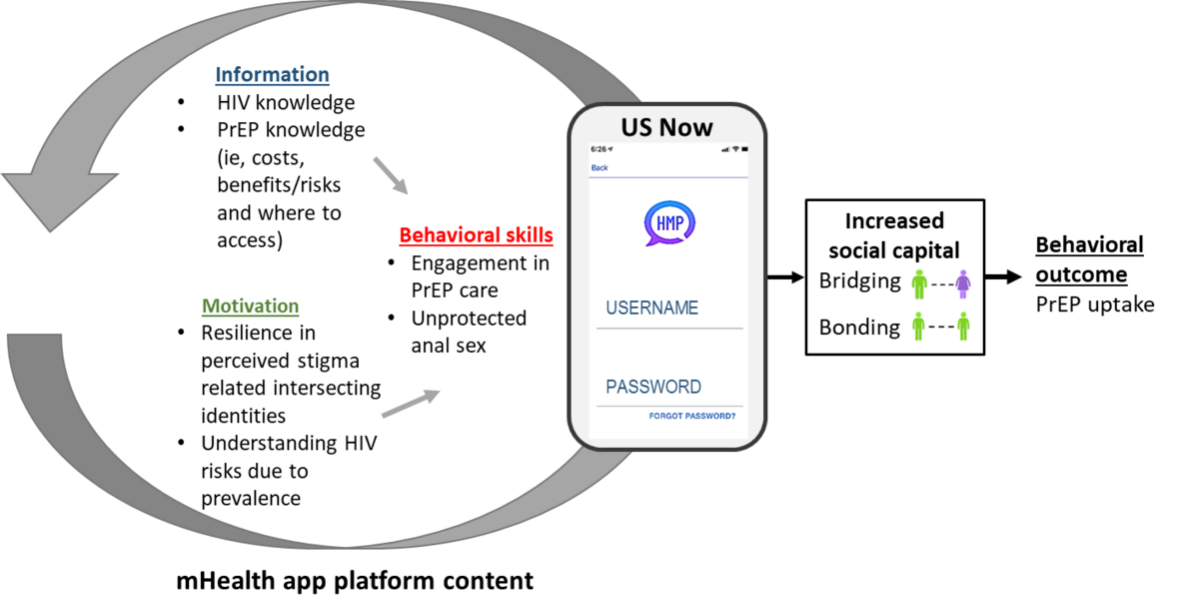
Adapted sIMB conceptual framework mapped to the PrEP US NoW intervention. Items in boxes are constructs of the Anderson Behavioral Model and sIMB, with arrows relating their interactions. mHealth: mobile health; PrEP: pre-exposure prophylaxis; PrEP US NoW: Pre-Exposure Prophylaxis Utilization Through Increasing Social Capital Among Young, Black, Sexual Minority Men Networks With Women; sIMB: situated Information, Motivation, and Behavioral skills.

This protocol paper has followed the SPIRIT (Standard Protocol Items: Recommendations for Interventional Trials) reporting guidelines (Multimedia Appendix 1) [39]. The theoretical foundation (Figure 1) for this study is an adaptation of the situated Information, Motivation, and Behavioral Skills (sIMB) model, which posits that information (information regarding PrEP use), motivation (personal and social attitudes and beliefs regarding sexual identity that facilitate PrEP use), and behavioral skills (objective and perceived skills, including self-efficacy, in accessing PrEP care) within the cultural and situational context in which they occur are necessary for behavioral change [40-42]. The adapted sIMB model is aimed at increasing PrEP use among young, Black, sexual minority men by increasing social capital within young, Black, sexual minority men’s social networks, by strengthening the bonds between young, Black, sexual minority men networks, and by gaining and having structured discussions around key topics known to influence PrEP use among this group (specifically, intersectional stigma, PrEP knowledge, and sexual health empowerment). We hypothesize that through these structured discussions, social network members will be able to gain key instrumental and emotional gains in social capital. Social networks yield social capital, which has health-promoting effects for individuals living with and at-risk for HIV. For this study, social capital was defined similar to Pierre [28], as the sum of an individual’s resource-containing, reciprocal, and trustworthy social network connections.

**Figure 2:**
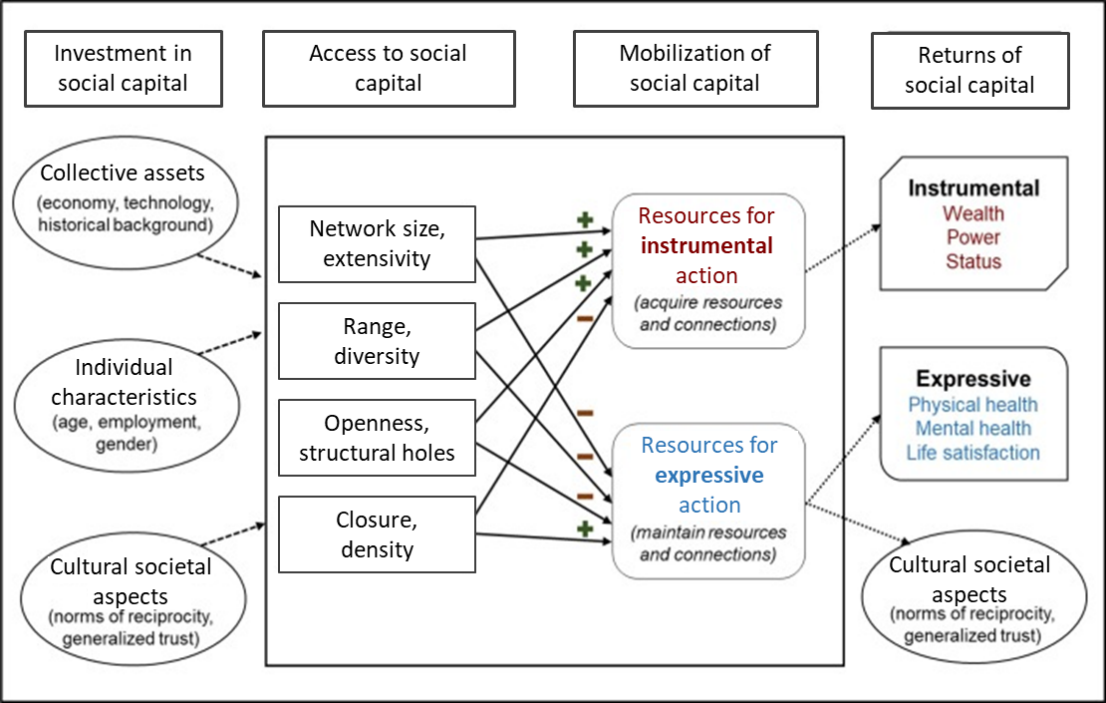
Potential factors and pathways for building social capital.

Figure 2 provides various pathways that are thought to impact the social capital within an individual, here young, Black, sexual minority men. For example, factors such as age (individual characteristic) can be considered as “investments in social capital,” which have a direct impact on the “access to social capital,” such as network size, which then builds a foundation for the “mobilization of social capital,” such as resources for instrumental gains, leading to “return of social capital,” such as power or status. Previously, sIMB has been used to improve PrEP adherence (iPrEx study), for studying engagement in HIV care, adherence to antiretroviral therapy, and HIV risk behavior change in adolescents [42-44]. However, to our knowledge, sIMB has never been applied to an intervention aimed at improving PrEP engagement by increasing social capital.

### Community Advisory Board

Initially, 2 community advisory boards (CABs), each consisting of 6 to 8 individuals, were proposed: one CAB composed of young, Black, sexual minority men and the other of Black women. The principle for the development of CABs was cocreation based on the principles of community-based participatory research [45]. However, based on feedback from potential CAB members, one combined group was created instead to mirror the goal of the study, allowing for social connectedness among Black, sexual minority men (BSMM; n=3) and Black women (n=5). The CAB members meet remotely and in-person at least monthly for 1 hour, and 1 Black female and 1 Black, sexual minority man serve as coleads. Depending on agenda topics, some meetings have included the principal investigators and some have been exclusive to CAB members, depending on whether topics discussed were felt to be sensitive. The CAB coleads were tasked with leading discussions on meeting agenda topics and facilitating direct communication with the investigative team for meetings where they were not present. Topics for the study reviewed with the CAB included study design, intervention content, app features, intervention beta testing, and recruitment procedures. Additionally, the CAB provided feedback on initial qualitative results and will provide feedback on the results from the pilot trial, which will assist with the dissemination of study findings.

### Designing the PrEP US NoW Intervention

#### Overview

In addition to regular discussions with the CAB, qualitative in-depth interviews were conducted with young, Black, sexual minority men and Black women. Through the interviews, a more granular understanding was gained of (1) young, Black, sexual minority men’s perceptions on the content needed to motivate engagement in the PrEP care continuum, (2) specific attributes of Black women that allow them to facilitate creation of a safe space, and (3) Black women’s perceptions of educational materials around safer sex promotion strategies that will be critical to enhance their abilities to be conduits of support when leading discussions for young, Black, sexual minority men’s social networks.

#### Recruitment and Sampling

Recruitment of young, Black, sexual minority men occurred in Birmingham, AL, through established connections of the principal investigators (LE in the Birmingham, AL, area and SH in Atlanta, GA); LE and SH have previous experience with recruiting young, Black, sexual minority men for other research studies. When young, Black, sexual minority men were screened in prior studies, respondents were able to indicate if they would like to be contacted for future studies, creating a contact list of HIV-negative young, Black, sexual minority men who are interested in being involved in research. In addition to contacting prior participants, flyers were posted to listservs and physical locations where young, Black, sexual minority men are known to socialize or seek medical care. In addition, the team recruited participants working with Black, sexual minority men study recruiters with large social followings and influence in the community. The eligibility criteria for young, Black, sexual minority men participants were (1) age 18-29 years, (2) cisgender men (based on gender and sex at birth), (3) self-reported past sex with other men (in the past 6 months), (4) Black race, and (5) English speaking. Of note, young, Black, sexual minority men already on PrEP were not excluded. A purposive sampling scheme was used to ensure representation of diverse perspectives of young, Black, sexual minority men. To include both younger and older young, Black, sexual minority men, half of the participants were 18-24 years old, and the other half were 25-29 years old.

Black women for in-depth interviews were recruited by direct referral by young, Black, sexual minority men or CAB members, or from prior studies. This was thought to ensure enrollment of Black women with close connections to young, Black, sexual minority men. Eligibility criteria for Black female participants included (1) Black race, (2) female (cisgender or transgender women), (3) having at least one close connection to a young, Black, sexual minority man, (4) age ≥18 years, and (5) English speaking. Since the aim of these interviews was to explore if certain traits, for example, age, made Black women more suited to lead the discussions, a specific age group within the Black women was not targeted.

#### In-Depth Interviews

Potential participants (young, Black, sexual minority men and Black women) were asked to complete a web-based screening tool to determine their eligibility. Once an individual was deemed eligible, they were contacted by the study staff via phone to schedule an in-depth interview. When in-person interviews were not possible, phone-based interviews were conducted. A semistructured interview guide was created with help from the CAB and refined through repeated pilot testing and revision. The interview guide (Table 1) consisted of a mixture of descriptive or narrative questions and questions structured around the domains of the adapted sIMB model, which, in addition to exploring participants’ experiences, also allowed for novel concepts and ideas to emerge. In-depth interviews were conducted in a private and quiet space mutually convenient for the interviewer (research coordinator or graduate research assistant) and the interviewee. During the interviews, interviewers documented field notes. All interviews were digitally audio recorded, deidentified, transcribed verbatim, and uploaded to an encrypted password-protected computer for the purpose of qualitative analysis.

**Table 1:** Interview guide associated with the PrEP US NoW^a^ study.

Topics			Operationalized topics
**Young, Black, sexual minority men (n=15)**			
	Information	Social support networks (types of relationships with young, Black, sexual minority men and Black women) Intersectional stigma (discussion of coping with discrimination, poverty, and sexual identity) addressed within social support networks	
	Motivation	Archetypical Black women who provide support Current social norms Types of interpersonal relationships that support HIV prevention uptake	
	Behavioral skills	Skills related to self-efficacy in navigating PrEPb care and condom use	
**Black women (n=20)**			
	Information	Information topics necessary to support young, Black, sexual minority men in sexual health Current attitudes and beliefs regarding young, Black, sexual minority men	
	Motivation	What would support Black women being engaged in young, Black, sexual minority men's social support networks	
	Behavioral skills	Skills required to counsel young, Black, sexual minority men on sexual health and HIV prevention	

^a^PrEP US NoW: Pre-Exposure Prophylaxis Utilization Through Increasing Social Capital Among Young, Black, sexual minority Men Networks With Women.

^b^PrEP: pre-exposure prophylaxis.

#### Qualitative Data Analysis

The coding and analysis of the transcripts were conducted using the NVivo qualitative data management software (QSR International). A preliminary codebook was developed, which consisted of (1) predetermined deductive codes related to sIMB domains of interest, (2) inductive codes emerging from the data, and (3) pattern codes connecting concepts to one another. The coding team applied these codes to a subset of transcripts, comparing coding strategies and adjusting code definitions until adequate intercoder reliability was achieved. During this process, the team also developed analytic memos regularly, reflecting dimensions and characteristics of each code, as well as noting major themes. Additionally, data matrices were created to visually represent relationships between key concepts and elicit patterns in the data. Throughout this process, the qualitative team held weekly analysis meetings (via Zoom) to facilitate collaboration between team members. Once the analysis was completed, findings were reviewed with the CAB as a form of member checking.

#### Translation of Qualitative Findings to Design the PrEP US NoW Intervention

Working with the CAB, the intervention mapping techniques [46] guided translation of nascent literature regarding barriers to PrEP use among young, Black, sexual minority men into an intervention protocol for subsequent piloting. Intervention mapping highlights the importance of incorporating data to map out the problem (ie, PrEP underuse by young, Black, sexual minority men), its determinants, and potential solutions. The planning team (CAB and study team) worked closely to describe the content for the web-based discussions by identifying the influences on PrEP uptake. Next, the team focused on the objectives, smaller goals, and the overarching larger goals for improving the PrEP care continuum. Moving forward, an intervention protocol was laid out, which informed the development of study materials, such as in-app discussion topics, training materials for female facilitators, etc, in a way that addressed the problems identified earlier. Next, the team worked to develop specific content, mapped to the sIMB conceptual framework, to facilitate web-based discussions around identified topics of importance. The team also worked with a technology partner to modify the app accordingly.

For this study, the existing HealthMpowerment (HMP) app was adapted, which would support online communities in discussions around stigma and sexual health [47-49]. The HMP app allows anonymity, which protects unintentional disclosure of HIV status and sexual identity and may help those facing HIV and sexual orientation stigma. This app also provides a virtual location for network members from different geographic locations, enabling them to discuss sensitive topics with shared access to information about HIV prevention strategies, like PrEP. Previous testing of the HMP app has shown that greater app engagement was significantly associated with stronger primary intervention effects (eg, condomless anal sex) and secondary effects such as HIV-related communication (eg, provider communication, HIV status disclosure to partners) and HIV care outcomes (eg, perceived barriers, engagement in care, self-reported adherence) [49-51].

The current participant forum in the HMP app was expanded to add a space where the intervention content will be delivered in real time through semistructured discussions led by Black female facilitators with the young, Black, sexual minority men network. Only Black women who possess the qualities identified in the qualitative findings associated with trust and nonstigmatizing support will lead the discussion topics. Black female facilitators and young, Black, sexual minority men network members will have the option of using an avatar to maintain confidentiality. At this time, the intervention mapping process with our CAB has led to the app name being changed to “US NoW.”

**Figure 3:**
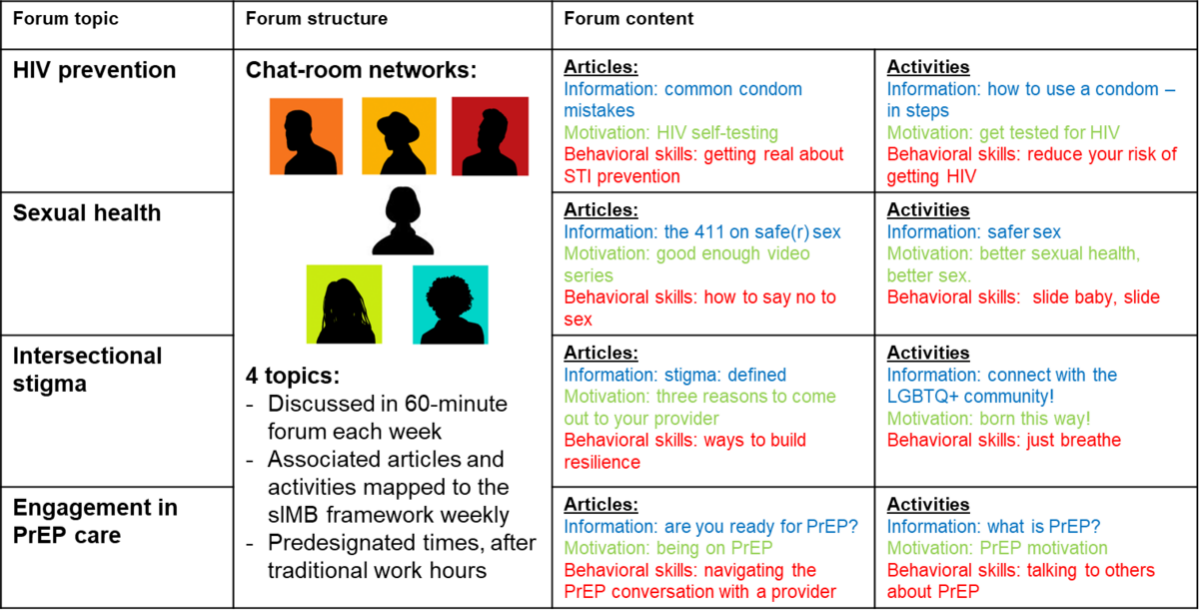
Community advisory board–informed topics, content, and structure for the intervention. Forum topics track the adapted sIMB conceptual framework. PrEP: pre-exposure prophylaxis; sIMB: situated Information, Motivation, and Behavioral skills; STI: sexually transmitted infection.

Each week, discussions ranging from 30 to 60 minutes will be conducted at a mutually agreed-upon time. Prior to each session, topic-specific scholarly articles suitable for the lay audience and activities will be provided in the HMP app that map the conceptual framework (see Figure 3). Young, Black, sexual minority men's social networks will be asked to view content and engage in discussions during these sessions. The young, Black, sexual minority men participants will be able to communicate and post privately as well as publicly with each other. If Black female leads are contacted ad hoc by a group member (between discussions or 1 week following the last discussion), they will be expected to reciprocate communication or request information within a 24-hour timeframe.

Beta testing of the app and intervention was carried out with the CAB to get a sense for how the features will work in the subsequent pilot trial. The CABs were oriented to the app and were asked to use it for a month. During the beta testing, 4 semistructured discussions were convened that mimicked how anticipated sessions will occur during the actual trial. The CAB members reconvened with the study team to provide feedback on their experiences, which was used to make the necessary final changes to the intervention prior to the randomized controlled trial.

#### Piloting and Evaluating the PrEP US NoW Intervention

**Figure 4:**
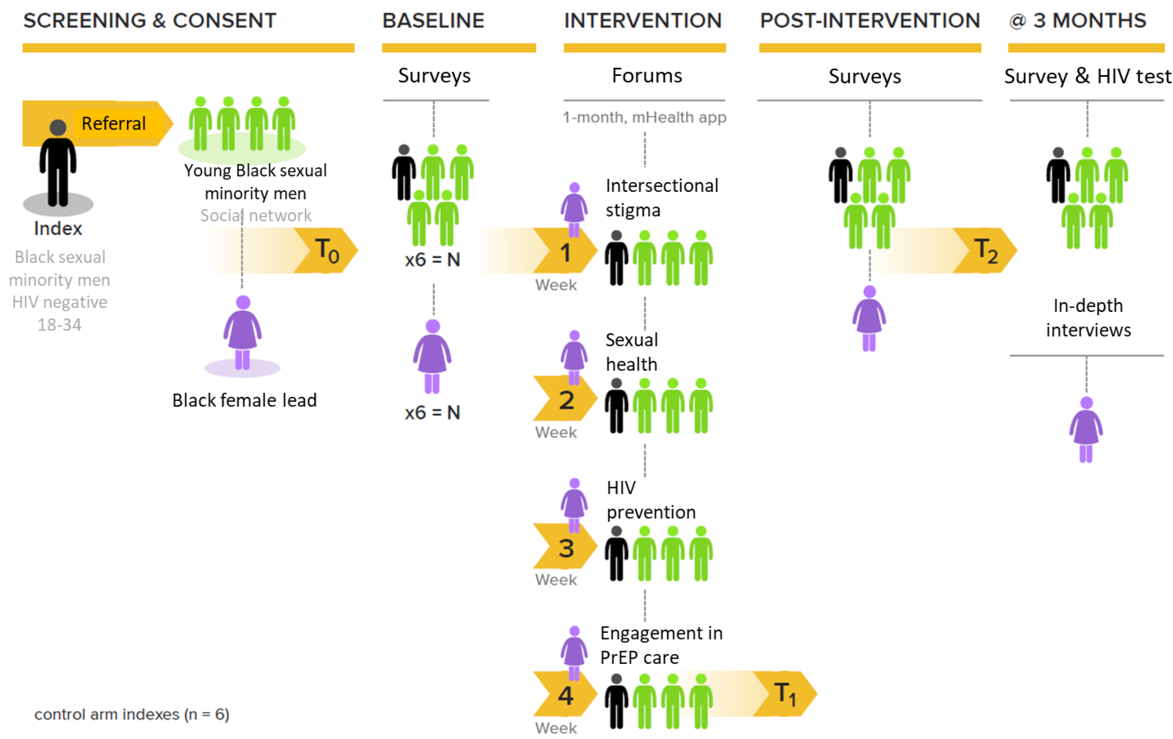
PrEP US NoW study design. One-to-one randomization will be done prior to this, with control arm indexes (n=10) to refer 8-10 young, Black, sexual minority men from their social network. These network referrals will be screened for eligibility, undergo sociograms, and then have access to the HealthMpowerment app alone, with surveys at T0, T1 (at 1 month), and T3 (at 3 months). mHealth: mobile health; PrEP: pre-exposure prophylaxis; PrEP US NoW: Pre-Exposure Prophylaxis Utilization Through Increasing Social Capital Among Young, Black, Sexual Minority Men Networks With Women.

The primary outcomes from the trial will include feasibility and acceptability, improvement in social capital, and reduction in internalized homonegativity. Secondary outcomes will include estimates of intervention efficacy in increasing PrEP uptake and decreasing condomless anal sex acts. Figure 4 outlines the study design for the pilot intervention.

#### Recruitment, Screening, and Consent

A network-based approach was used to identify social network members of the index young, Black, sexual minority men participants by following a sociogram protocol [52], that is, graphic plotting of the young, Black, sexual minority men’s social network members. The recruitment for young, Black, sexual minority men is still ongoing, whereas recruitment for Black female group facilitators has been completed. For each index young, Black, sexual minority man in the intervention arm, a Black female social network member, referred by the CAB and external to the young, Black, sexual minority men's network, was screened. The inclusion criteria for the Black women facilitators included (1) self-identified Black race and female gender, (2) age >18 years, and (3) fluency in written and spoken English. The goal was to have at least one eligible Black female member per 6 eligible young, Black, sexual minority men, serving as a facilitator in each group. The ultimate Black female facilitator selection only consisted of cisgender women and was made by the CAB based on a structured interview to assess stigmatizing beliefs and cultural humility. As this was an egocentric network analysis, the investigators assumed networks were independent of one another; however, the first name and last initial of participants were collected to evaluate for overlaps across networks in the analyses.

To recruit networks of young, Black, sexual minority men, index young, Black, sexual minority men participants will be recruited first, who will subsequently recruit up to 8 young, Black, sexual minority men members of their own social networks. Similar resources and strategies used earlier will be used to recruit index young, Black, sexual minority men participants. Inclusion criteria for young, Black, sexual minority men index participants are (1) age 18-29 years, (2) HIV negative (based on self-performed HIV OraQuick test), (3) Black cisgender men (based on gender and sex at birth), (4) self-reported past sex with other men (past 6 months), (5) English speaking, and (6) able to identify 6-8 young, Black, sexual minority men in their personal social network. The exclusion criterion is cognitive impairment resulting in the inability to provide consent. Potential index participants will complete a web-based screening survey to determine eligibility. Once an individual is considered eligible, they will be contacted (via phone or in person) to provide more details about the study and will be asked to complete an informed consent. Enrolled index young, Black, sexual minority men participants (n=12) will be randomized (2:1) to the intervention arm (n=30, 6 indices with 5 network members per index) or the control arm (n=30, 6 indices with 5 network members per index). The allocation sequence is generated and assigned by REDCap (Research Electronic Data Capture), and participants are enrolled by our research coordinator. The allocation is computer-generated for randomization by our REDCap. Allocation concealment is provided by the REDCap system. Participants' IDs are provided to our data manager, who enters this information into REDCap. They then inform our research coordinator of the randomization arm for the participant. Both the intervention and control arms will be unblinded. At recruitment, the participants will be educated on the protocol and goal of the study, understanding how support systems can improve access to HIV prevention strategies like PrEP. The randomized controlled trial was registered within ClinicalTrials.gov (NCT07024745).

#### Intervention Arm

A total of 6 young, Black, sexual minority men's social networks, each including 5 young, Black, sexual minority men (including index participants) and 1 Black female facilitator, will participate in the intervention arm. Before the trainings, the Black female facilitators completed a baseline survey electronically, which measured sociodemographics, HIV knowledge, PrEP knowledge, HIV-related stigma [53], homophobia [54], and PrEP stigma. Black female facilitators have completed their training on app usage. In addition, Black females also completed an (web-based or face-to-face) intensive training covering (1 day) cultural competence and humility when working with sexual and gender minority populations; experiences of stigma among young, Black, sexual minority men in the South; LGBTQ-focused sexual health topics; and basic information about HIV treatment and prevention, including the use of PrEP and steps required to engage in PrEP care.

All young, Black, sexual minority men in the intervention arm will also receive a training (web-based or face-to-face), including a tour of the downloadable US NoW app, a brief demonstration of how to join and participate in the web-based discussion, and practice on posting content publicly and privately. Once trained, young, Black, sexual minority men will complete HIV testing using OraQuick rapid HIV test (remote participants will be asked to text a picture of the test result via a secure server) and an electronic baseline survey prior to study commencement capturing information on sociodemographics, stigma related to race or ethnicity and sexual practices or orientation [55,56], HIV, poverty, PrEP use [57], PrEP knowledge [57], sexual history and practices [58], alcohol [59] and substance abuse [60], and depression [61] and anxiety [62], using appropriate validated measures. In the event an individual tests positive on an HIV test, they will be referred to the linkage team at the study site’s university-affiliated clinic for counseling, access to confirmatory testing, and linkage to care, if necessary.

Following enrollment of young, Black, sexual minority men and Black women facilitators, 4 predesignated 60-minute discussions with the network and the Black woman facilitator will commence. Activities of Black female facilitators will include leading discussions and answering any questions or comments posted by young, Black, sexual minority men. Young, Black, sexual minority men participants will be instructed how to use the app throughout the 4-week period. The importance of attendance at the discussions will be emphasized and incentivized through app badges given by other members within the network and by Black female facilitators. Additionally, a reminder, through phone texts, will be sent to young, Black, sexual minority men participants about discussion times.

The data collection began in August 2024 and is expected to be completed in December 2025. Once the intervention ends, young, Black, sexual minority men participants will receive follow-up surveys electronically at *T*_1_ (1-month postintervention) and *T*_3_ (3-month postintervention), capturing the primary and secondary outcomes (explained in detail later). Young, Black, sexual minority men will also be asked to perform a rapid HIV OraQuick test at *T*_3_, remotely. Similarly, Black female facilitators will also complete a follow-up electronic survey at *T*_1,_ which will measure feasibility and acceptability, as well as any changes in stigma and PrEP knowledge or attitudes from baseline. In addition to surveys, Black female facilitators will also participate in web-based qualitative interviews at *T*_3_, which will evaluate PrEP knowledge, intersectional stigma, and change in attitudes following the intervention and gain descriptive information about the process (ie, feasibility and acceptability) of PrEP US NoW facilitation. Additionally, experiences and comfort around delivering the intervention will also be assessed.

#### Control Arm

The control arm will be similar to the intervention arm in terms of HIV testing, baseline and follow-up surveys, and incentives. However, there will be no Black female facilitators, and instead of the modified intervention HMP app, these participants will use a control version of the HMP app without any discussions or scheduled check-ins with study staff. These participants will, however, be provided with the opportunity to contact the study team in case of technical difficulties or any other issues.

#### Survey Measures

##### Independent Variables

Using validated measures, the following will be assessed: sociodemographics (age, income, employment, education, insurance, and housing); stigma related to race or ethnicity, sexual practices or orientation, HIV, poverty, and PrEP; PrEP knowledge; sexual history and practices; alcohol and substance abuse; and depression and anxiety.

##### Primary Outcomes

Four primary outcomes—feasibility, acceptability, social capital, and homophobia—will be measured. *Feasibility* and *acceptability* will be measured using responses from *T*_3_ surveys. Validated scales will include the Acceptability of Intervention Measure, Intervention Appropriateness Measure, and Feasibility of Intervention Measure [63]. These scales include straightforward questions such as “[PrEP US NoW] is appealing to me,” to which participants respond on a 5-point Likert scale. Feasibility will also be measured by monitoring in-app usage characterized by the completion of 15-minute discussions, the number of posts, and the total time spent in virtual discussions. Moreover, intervention fidelity will be monitored by documenting the completion of 15-minute discussions and the response rate for questions posted, and by reviewing the accuracy of information shared. *Social capital* will be measured using a modified version of Chen’s Personal Social Capital, the Modified Social Capital Scale [64], adapted for young, Black, sexual minority men use. This scale measures bonding (resources accessed within similar sociodemographic social groups) and bridging (resources derived from cross-identity connections) social capital with sources and types of support (eg, emotional, instrumental, and informational). *Homophobia* will be measured using the Internalized Homophobia Scale [65].

##### Secondary Outcomes

The secondary outcomes will include *connectedness to one’s own ethnic community,* measured using an adapted version of the Social Connectedness in the Ethnic Community Scale [66], *condomless anal intercourse in the past 3 months* (ordinal measures), and *PrEP uptake* (yes or no).

##### Sample Size Justification

For this pilot study, the importance of sample size lies in establishing feasibility and the precision of estimated changes, which will inform the design of future studies. Twelve index participants will be recruited, and each index will provide information to recruit 4 others, yielding a total sample size of 60. Six indexes will be randomized to PrEP US NoW and 6 to the control group. The clustering of participants within the index (specifically the PrEP US NoW group) may result in a reduced effective sample size. Assuming no correlation within indices for all participants, a rate of advancement of 50% for PrEP US NoW, the bootstrap 95% CI would have a margin of error of ±12%. On the other hand, assuming a large correlation, 0.9, the margin of error becomes ±25%. With an anticipated 15% PrEP uptake, the 95% CI margin of error is ±9% for no correlation and ±18% for a correlation of 0.9.

### Data Analysis

The data will be stored in a secure server housed in the health system at the University of Alabama at Birmingham and will only be viewed on designated encrypted devices by the principal investigator and study team. Descriptive statistics will be calculated to characterize acceptability and feasibility measures. Stratifying young, Black, sexual minority men by age ranges, 18-21 and 22-29 years, all outcomes will be described by independent variables using frequencies (percentages). Generalized estimated equations will be used to perform logistic regression with empirical standard errors using an exchangeable working correlation, based on the reasonable assumption of equal correlation among members within each index. Other structures will be examined. These models will be used to estimate the rate of PrEP uptake to determine the difference in odds of PrEP uptake, controlling for within-index correlation to determine the difference in the odds of uptake between the two groups (intervention and control). Separate models will be fitted directly after the intervention (*T*_1_) and 3 months following the intervention (*T*_3_). For each model, independent predictor variables (sociodemographic factors, variables related to stigma, knowledge, sexual history and practices, alcohol and substance abuse, and depression and anxiety) will be included as covariates. All categorical outcomes (advancement, change in social capital, connectedness, and condomless anal intercourse in the past 3 months) will be treated similarly. Lastly, if there are overlaps in social networks between index cases, we will perform a network analysis to determine closeness, betweenness, and degree to determine if there will be potential for treatment interference.

### Data Management

Results of remotely conducted HIV tests will be shared via a secure server hosted in the university’s health system. The results will only be viewed by the study staff on encrypted mobile devices. All electronic surveys will be entered into a secure, web-based application, REDCap. For quality control purposes, REDCap has data visualization tools to assist in data cleaning and evaluation. The Data Export Tool includes methods for data deidentification. The REDCap database is hosted at the UAB Department of Medicine’s secure data center, which will be used as a central location for data storage, processing, and management. The servers are protected by an aggressive firewall and a log monitoring system. All web communications are protected via SSL encryption. Only institutional review board (IRB)–approved research team members will have access to the REDCap. Access is granted according to “the principle of least privilege.” Each team member will be granted access to the REDCap data system through a secure log-in. The data will be deidentified for analysis, and findings will be disseminated in an aggregate form. All digital files will be deleted, and all physical files will be shredded after 5 years in line with the University of Alabama at Birmingham’s policy.

### Ethical Considerations

This study was approved by the University of Alabama at Birmingham’s institutional review board (IRB-300007813) on October 18, 2021. Informed consent was collected and documented from all young, Black, sexual minority men and Black female participants (Multimedia Appendices 2 and 3). The qualitative and quantitative data used for this study were covered under the study’s IRB. The consent forms were documented electronically. All data were deidentified before being analyzed. All CAB members were compensated with US $50 per meeting. Black women in the intervention arm underwent a training and received US $400 after training completion. In addition, young, Black, sexual minority men and Black women will complete 3 surveys (baseline, *T*_1_, and *T*_3_) and will be incentivized with US $50 per survey.

## Results

For informing the PrEP US NoW intervention, 52 individuals (young, Black, sexual minority men: 33; Black women: 19) completed a web-based screening, which lasted between May 2022 and June 2024. Of these, a total of 30 were selected for semistructured interviews (young, Black, sexual minority men: 15; Black women: 15). The interviews were conducted between May 2022 and June 2024. The findings from the interviews indicated that young, Black, sexual minority men value emotional support the most and that Black women provide both emotional and tangible support (manuscript in the process of submission). These findings were then applied in designing the app for the intervention. The intervention is currently in the initial phases of recruitment. The intervention arm will have 6 social network groups, each comprising 5 young, Black, sexual minority men and 1 Black female facilitator. The control arm is expected to have 30 young, Black, sexual minority men participants. The baseline surveys with young, Black, sexual minority men began in August 2024 and are still ongoing. The baseline surveys with Black females were completed between July and August 2024. The young, Black, sexual minority men's follow-up surveys at *T*_1_ and *T*_3_, along with Black women’s surveys at *T*_1_ and qualitative interviews at *T*_3_, are expected to be completed by the end of 2025. The analysis of findings from young, Black, sexual minority men and Black women in the intervention arm and young, Black, sexual minority men in the control arm will be helpful in assessing the effectiveness of the PrEP US NoW intervention in improving PrEP uptake among young, Black, sexual minority men. If the study shows promising findings, a subsequent grant application will be submitted, which will help develop a fully powered randomized controlled trial that can test the efficacy of the intervention at multiple sites across the US South. In case the findings are not as anticipated, the knowledge gained on young, Black, sexual minority men's social networks, social capital, and the role of Black women facilitators will still provide novel insights to inform future HIV prevention research among this highly impacted group.

## Discussion

### Anticipated Findings

This paper describes a novel research protocol that will develop and pilot an mHealth intervention, PrEP US NoW, which aims to improve engagement in PrEP care among young, Black, sexual minority men in Birmingham, AL, by leveraging and supporting the social capital within their social networks. This intervention is anticipated to increase social capital in accessing sexual health services, like PrEP, among young, Black, sexual minority men. The pathways by which this is thought to occur are related to increasing knowledge around sexual health well-being, specifically PrEP. In addition, by focusing on motivating participants in engaging in the sexual health system, self-efficacy in accessing sexual health services as an empowered self-advocate will also increase. With a high HIV incidence rate among young, Black, sexual minority men in the Southern United States, coupled with a low PrEP-to-need ratio among this population [20], the PrEP US NoW intervention is timely in that it addresses this disparity effectively among young, Black, sexual minority men, a group at significant risk for HIV, by evaluating strategies to increase uptake of PrEP, a highly efficacious HIV prevention tool. The PrEP US NoW intervention hence aligns with the national priorities outlined by the EHE initiative.

Previously, a study used social networks to improve PrEP uptake among young, Black, sexual minority men [36]. In this study, each young, Black, sexual minority men participant was asked to identify and recruit a social confidant in the study. As a part of the intervention, the young, Black, sexual minority men and their social confidant met with the social worker 4 times to develop a retention in care plan. The goal of these meetings was to improve HIV-care knowledge and to activate the dyadic social support in order to improve PrEP uptake. With the control group receiving the usual treatment without a social confidant, the participants in the intervention group showed significantly better retention in care outcomes than the controls, particularly kept-visits. Similarly, another study enrolled 5 HIV-negative Black, sexual minority men, who then involved members of their social networks, who were predominantly Black [24]. These social groups then engaged in weekly group sessions and booster sessions led by the project’s staff. The topics discussed in these sessions involved improving knowledge on PrEP, its importance in HIV prevention, and the process of initiating and maintaining PrEP use. This study showed an increase in PrEP knowledge, PrEP use willingness, and PrEP use prevalence after the intervention. While these studies showed positive PrEP uptake outcomes, they mainly engaged network members with similar sociodemographics to the index participant, for example, young, Black, sexual minority men getting help from young, Black, sexual minority men peers. Additionally, while one of these studies also identified Black women as trusted members of young, Black, sexual minority men’s social networks [36], there is a dearth of research evaluating the role of Black women within social networks for young, Black, sexual minority men or leveraging these bonds to improve health outcomes. Keeping these gaps in mind, an innovative approach was designed, which will use a network-based approach by involving both young, Black, sexual minority men and Black women (considered trustworthy by young, Black, sexual minority men) to engage in discussions on intersectional stigma, sexual health, HIV or sexually transmitted infection prevention, and PrEP, which are expected to improve their social capital and subsequently PrEP uptake. Regardless of the intervention outcomes, the results from this intervention would enrich the current literature on the role of Black women figures in young, Black, sexual minority men's social networks in HIV prevention.

While the intervention is expected to be completed within the described timeline, in case young, Black, sexual minority men's networks are not reliably attending the web-based discussions, the intervention may get extended for 5-6 weeks. Although best practices were adopted to design and implement this intervention, there may be a few limitations. For example, there is a potential for selection bias, where young, Black, sexual minority men who are interested in participating in this study may have less internalized stigma than the general population of young, Black, sexual minority men. To avoid this, necessary efforts were made to recruit using web-based approaches and to recruit in rural settings where there was more stigma, leading to fear of unintentional disclosure of sexual orientation through participation in the intervention. Additionally, some young, Black, sexual minority men may be unstably housed or have other disruptions to research participation. To address this, multiple contact methods (email, phone, and social media) will be obtained, and this information will be updated every 3 months.

### Conclusions

The young, Black, sexual minority men living in the Southern United States are disproportionately impacted by the HIV epidemic. This population has a high incidence rate of HIV coupled with a low PrEP-to-need ratio. This intervention aims to increase social capital among young, Black, sexual minority men in gaining self-efficacy on their sexual health and navigating structural barriers to access PrEP services in Alabama (1 of the 11 non-Medicaid expansion states), ultimately leading to increased PrEP uptake among this population.

## Appendix

Multimedia Appendix 1 SPIRIT checklist.

Multimedia Appendix 2 Informed consent form: Black female participants.

Multimedia Appendix 3 Informed consent form: young, Black, sexual minority men participants.

## Abbreviations

CAB: community advisory board
EHE: Ending the HIV Epidemic
HMP: HealthMpowerment
IRB: institutional review board
mHealth: mobile health
PrEP: pre-exposure prophylaxis
PrEP US NoW: Pre-Exposure Prophylaxis Utilization Through Increasing Social Capital Among Young, Black, sexual minority Men Networks With Women
REDCap: Research Electronic Data Capture
sIMB: situated Information, Motivation, and Behavioral skills
SPIRIT: Standard Protocol Items: Recommendations for Interventional Trials

## Data Availability

The datasets generated during this study will be available from the corresponding author on reasonable request.
