# Supplementary material for: An mHealth App–Based Social Capital Intervention (PrEP US NoW) to Improve Sexual Health and Uptake of Pre-Exposure Prophylaxis Among Young, Black, Sexual Minority Men: Protocol for Intervention Development and a Pilot Randomized Controlled Trial
Source: JMIR Res Protoc. 2025 Sep 18;14:e66326. doi: 10.2196/66326 (PMC12491899; doi:10.2196/66326)
Supplement: Multimedia Appendix 2 [file resprot_v14i1e66326_app2.pdf]

CONSENT FORM TO BE PART OF A RESEARCH STUDY

Title of Research: PrEP US NoW: PrEP Utilization Through Increasing Social Capital Among YBMSM Networks with Women

UAB IRB Protocol #: IRB-300007813

Principal Investigator: Latesha E. Elopre, MD, MSPH

Sponsor: National Institutes of Mental Health

**General Information**	You are being asked to take part in a research study. This research study is voluntary, meaning you do not have to take part in it. The procedures, risks, and benefits are fully described further in the consent form.
**Purpose**	The purpose of the study is to improve engagement in HIV pre-exposure prophylaxis (PrEP) care among young, Black men who have sex with men (YBMSM) in the South, using Black women in the YBMSM social networks to help.
**Duration & Visits**	If you agree to be in this study, you will complete an enrollment visit where you will complete a survey, receive instructions on how to download the Us*NoW study app, and receive information for the training. The enrollment survey will take 45 to 60 minutes, and the instructions for the Us*NoW app and training will take 15 minutes.<br><br>The training will be a 1 day hybrid meeting. The purpose of this training is to prepare you to facilitate a group of YBMSM through the intervention. The topics that will be covered include Cultural Competence and Humility, Sexual Health, HIV Basics and Prevention, and Intervention and App Orientation. The training will be led by a member of the study staff. The training will take 7-8 hours.<br><br>The Intervention will consist of you leading a weekly group sessions with 5-6 YBMSM on topics assigned by study staff. There will be 4 total weekly group sessions, lasting 60 minutes each.<br><br>In addition to the enrollment survey, you will be asked to complete surveys at the end of the intervention (1-month) and 3-month post intervention. These surveys will also take 45 to 60 minutes to complete.<br><br>You will also complete an interview at the end of the intervention, asking your opinion on how it went.
**Overview of Procedures**	At the enrollment visit, you will download the Us*NoW study app, which is a health digital app that is designed specifically for this study that provides information and resources on PrEP and sexual health.<br><br>The surveys will be sent to you via email. If you do have an email address, study staff will arrange a time to do the survey over the phone with you.<br><br>The zoom link for the group sessions will be sent through the app messaging feature.

**Risks**	The most common risk include risk for breach of confidentiality and psychological distress.
**Benefits**	You may or may not benefit by being in the study. However, this study may help us understand how to better offer HIV prevention services in the future.
**Alternatives**	You can choose not to participate in the study.

Purpose of the Research Study

We are asking you to take part in a research study. The purpose of this study is to evaluate how effective and practical an intervention, led by a Black female, would be to increase engagement in HIV pre-exposure prophylaxis (PrEP) among young, Black men who have sex with men (YBMSM). PrEP is a once daily oral pill that can be taken to prevent infection with HIV after potential exposures to the virus. The information we learn from this study will help us develop behavioral interventions to help increase PrEP use in this population.

There will be 6 Black women participants enrolled in this part of the study.

Study Participation & Procedures

If you agree to join the study, you will be asked to facilitate a group (4-5) of young, Black men who have sex with men (YBMSM) through the intervention using the Us*NoW app.

Training

You will be asked to participate in a one time one-day, 7-8 hour hybrid training. The purpose of this training is to prepare you to facilitate a group of YBMSM through the intervention. We will ask you to join via Zoom ideally, but in-person space at the 1917 Clinic will be available for those who cannot join via Zoom. Talk to the study team member about which option works for you. All training materials will be sent to your house prior to the training. The topics that will be covered include Cultural Competence and Humility, Sexual Health, HIV Basics and Prevention, and Intervention and App Orientation. The training will be led by a member of the study staff.

Intervention

The intervention will last 4 weeks. You will be asked to interact with YBMSM in the group through the Us*NoW app using the messaging feature within the app. You will also lead 1-hour long weekly sessions with the group. During these weekly sessions, you will have weekly topics to discuss with the group. Materials will be given prior to intervention start.

As part of this study you will interact and engage with the Us*NoW app, which is a digital app designed specifically for YBMSM that provides information and resources on PrEP and other sexual health topics. In the app, YBMSM participants will be able to track their medications, read articles, complete informative quizzes and assessments, track behaviors (e.g., smoking, healthy eating, mood, sex), and find additional health resources based off their location or services needed.

Survey

You will complete 3 surveys during your time in this study at the following timepoints: Prior to training, Immediately following the intervention, and 3 month post intervention. The survey's will be completed on REDCap and will be emailed to you or you can complete over the phone with a study staff member.

Interview
You will be invited to complete an interview at the end of the intervention. We will ask you what your thoughts were on leading the YBMSM group and thoughts on the training materials. We will audio record the interview and it should take 1 hour to complete.

Risks and Discomforts

As with all research, there is a risk for breach of confidentiality. We try to minimize this risk by keeping your information private and secured, available to those involved with this study trained to keep information confidential, assigning a unique code to your study records instead of using your name or other identifiers on the research data we collect, and password-protecting any electronic research data.

Benefits

You may not benefit directly from taking part in this study.

Alternatives

You can choose not to participate in the study.

Confidentiality

Information obtained about you for this study will be kept confidential to the extent allowed by law. However, research information that identifies you may be shared with people or organizations for quality assurance or data analysis, or with those responsible for ensuring compliance with laws and regulations related to research. They include:

- the UAB Institutional Review Board (IRB). An IRB is a group that reviews the study to protect the rights and welfare of research participants.
- National Institute of Mental Health (NIMH)
- the Office for Human Research Protections (OHRP)

The information from the research may be published for scientific purposes; however, your identity will not be given out in those publications.

This research is covered by a Certificate of Confidentiality from the National Institutes of Health. The researchers with this Certificate may not disclose or use information, documents, or biospecimens that may identify you in any federal, state, or local civil, criminal, administrative, legislative, or other action, suit, or proceeding, or be used as evidence, for example, if there is a court subpoena, unless you have consented for this use. Information, documents, or biospecimens protected by this Certificate cannot be disclosed to anyone else who is not connected with the research except, if there is a federal, state, or local law that requires disclosure (such as to report child abuse or communicable diseases but not for federal, state, or local civil, criminal, administrative, legislative, or other proceedings, see below); if you have consented to the disclosure, including for your medical treatment; or if it is used for other scientific research, as allowed by federal regulations protecting research subjects.

The Certificate cannot be used to refuse a request for information from personnel of the United States federal or state government agency sponsoring the project that is needed for auditing or program evaluation by the *NIMH* which is funding this project or for information that must be disclosed in order to meet the requirements of the federal Food and Drug Administration (FDA). You should understand that a Certificate of Confidentiality does not prevent you from voluntarily releasing information about yourself or your involvement in this research. If you want your research information released to an insurer, medical care

provider, or any other person not connected with the research, you must provide consent to allow the researchers to release it.

The Certificate of Confidentiality will not be used to prevent disclosure as required by federal, state, or local law of, such as child abuse and neglect, or harm to self or others.

The Certificate of Confidentiality will not be used to prevent disclosure for any purpose you have consented to in this informed consent document.

Voluntary Participation and Withdrawal

Whether or not you take part in this study is your choice. There will be no penalty if you decide not to be in the study. If you decide not to be in the study, you will not lose any benefits you are otherwise owed. You are free to withdraw from this research study at any time. Your choice to leave the study will not affect your relationship with this institution. Contact the study doctor or staff if you want to withdraw from the study.

Cost of Participation

There will be no cost to you for taking part in this study.

Payment for Participation

You will be paid $200 for attending the full day training. You will be paid $50 for each weekly session completed. You will be paid $30 for each survey completed, and $50 for completing the interview at the end of the intervention. Total amount you will receive for participation is $540. Ask the study staff about the method of payment that will be used for this study (e.g., check, cash, gift card, direct deposit).

You are responsible for paying any state, federal, Social Security or other taxes on the payments you receive. You will receive a form 1099 in January of the year following your participation in this study. This form is also sent to the IRS to report any money paid to you. No taxes are kept from your payment.

Optional Research

Our research group (RISC- Research and Informatics Service Center) conducts many research studies. We would like the opportunity to contact you for future studies you may be eligible to participate in. You may change your mind at any time.

Initial your choice below:

_____ I agree to be contacted for future research studies in RISC.

_____ I do NOT agree to be contacted for future research studies in RISC

New Findings

You will be told by the study doctor or the study staff if new information becomes available that might affect your choice to stay in the study.

Questions

If you have any questions, concerns, or complaints about the research, please contact the study doctor. You may contact Dr. Latesha Elopre at 205-975-2457.

If you have questions about your rights as a research participant, or concerns or complaints about the research, you may contact the UAB Office of the IRB (OIRB) at (205) 934-3789 or toll free at 1-855- 860-3789. Regular hours for the OIRB are 8:00 a.m. to 5:00 p.m. CT, Monday through Friday.

Legal Rights

You are not waiving any of your legal rights by signing this consent form.

Signatures

Your signature below indicates that you have read (or been read) the information provided above and agree to participate in this study. You will receive a copy of this signed consent form.

____

Signature of Participant Date

____

Signature of Person Obtaining Consent Date
