# Supplementary material for: An mHealth App–Based Social Capital Intervention (PrEP US NoW) to Improve Sexual Health and Uptake of Pre-Exposure Prophylaxis Among Young, Black, Sexual Minority Men: Protocol for Intervention Development and a Pilot Randomized Controlled Trial
Source: JMIR Res Protoc. 2025 Sep 18;14:e66326. doi: 10.2196/66326 (PMC12491899; doi:10.2196/66326)
Supplement: Multimedia Appendix 3 [file resprot_v14i1e66326_app3.pdf]

UAB IRB
Approved
27-Nov-2023
until
23-Oct-2025

CONSENT FORM TO BE PART OF A RESEARCH STUDY

Title of Research: PrEP US NoW: PrEP Utilization Through Increasing Social Capital Among YBMSM Networks with Women

UAB IRB Protocol #: IRB-300007813

Principal Investigator: Latesha E. Elopre, MD, MSPH

Sponsor: National Institutes of Mental Health

**General Information**	You are being asked to take part in a research study. This research study is voluntary, meaning you do not have to take part in it. The procedures, risks, and benefits are fully described further in the consent form.
**Purpose**	The purpose of the study is to improve engagement in HIV pre-exposure prophylaxis (PrEP) care among young, Black men who have sex with men (YBMSM) in the South, using Black women in the YBMSM social networks to help.
**Duration & Visits**	If you agree to be in this study, you will complete an enrollment visit where you will complete a survey, receive instructions on how to download the Us*NoW study app, and receive a HIV self-test kit with instructions to be conducted at home. The enrollment survey will take 45 to 60 minutes to complete and the instructions to download the app will take 15 minutes to complete.<br><br>If you are randomized to the Intervention arm, you will participate in 4 weekly 60-minute virtual sessions, via Zoom, with a group of 4-5 other young, Black men who have sex with men (YBMSM). The session will be facilitated by a black female peer. You will be asked to interact with the Us*NoW app for 4 weeks. There will be weekly articles and activities highlighted in the app, in addition to other PrEP and sexual health resources. We will conduct interviews with a few participants from this arm at the end of the 4-weeks, asking their thoughts on the app and group sessions. Those interviews will be audio recorded and will take 60 minutes to complete.<br><br>If you are randomized to the Control arm, you will ask be asked to interact with the Us*NoW app for 4 weeks. There will be weekly articles and activities highlighted in the app, in addition to other PrEP and sexual health resources. We will conduct interviews with a few participants from this arm at the end of the 4-weeks, asking their thoughts on the app. Those interviews will be audio recorded and will take 60 minutes to complete.<br><br>In addition to the enrollment survey, you will be asked to complete surveys at the end of the intervention (1-month) and 3-month post intervention. These surveys will also take 45 to 60 minutes to complete.
**Overview of Procedures**	At the enrollment visit, you will download the Us*NoW study app, which is a health digital app that is designed specifically for this study that provides

	information and resources on PrEP and sexual health.<br><br>The surveys will be sent to you via email. If you do have an email address, study staff will arrange a time to do the survey over the phone with you.<br><br>If you are in the Intervention arm, the zoom link to the group sessions will be sent through the app messaging feature.
**Risks**	The most common risk include risk for breach of confidentiality and psychological distress.
**Benefits**	You may or may not benefit by being in the study. However, this study may help us understand how to better offer HIV prevention services in the future.
**Alternatives**	You can choose not to participate in the study, and this will not affect your medical care or opportunities for care coordination in any way.

Purpose of the Research Study

We are asking you to take part in a research study. The purpose of this study is to conduct an intervention, led by a Black female, would be to increase engagement in HIV pre-exposure prophylaxis (PrEP) among young, Black men who have sex with men (YBMSM). PrEP is a once daily oral pill that can be taken to prevent infection with HIV after potential exposures to the virus. The information we learn from this study will help us develop behavioral interventions to help increase PrEP use in this population.

There will be 60 YBMSM participants enrolled in this part of the study.

Study Participation & Procedures

If you agree to join the study, you will participate in a 4-week app based intervention with approx. 4-5 other young, Black men who has sex with men (YBMSM) participants.

Enrollment

You will complete an enrollment visit where you will complete a survey, receive instructions on how to download the Us*NoW study app, and complete a HIV self-test kit. You will share those test results with study staff during the enrollment visit. . The enrollment survey will take 45 to 60 minutes to complete and the instructions to download the app will take 15 minutes to complete. Additionally, you will be randomized to either the Control or Intervention arms.

Intervention

If you are randomized to the Intervention arm, you will participate in 4 weekly 60-minute virtual sessions, via Zoom, with a group of 4-5 other YBMSM. The session will be facilitated by a black female peer. You will be asked to interact with the Us*NoW app for 4 weeks. There will be weekly articles and activities highlighted in the app, in addition to other PrEP and sexual health resources. We will conduct interviews with a few participants from this arm at the end of the 4-weeks, asking their thoughts on the app and group sessions. Those interviews will be audio recorded and will take 60 minutes to complete.

If you are randomized to the Control arm, you will ask be asked to interact with the Us*NoW app for 4 weeks. There will be weekly articles and activities highlighted in the app, in addition to other PrEP and sexual health resources. We will conduct interviews with a few participants from this arm at the end of the 4-weeks, asking their thoughts on the app. Those interviews will be audio recorded and will take 60 minutes to complete.

As part of this study you will interact and engage with the Us*NoW app, which is a digital app designed specifically for YBMSM that provides information and resources on PrEP and other sexual health topics. In the app, you will be able to track your medications, read articles, complete informative quizzes and assessments, track behaviors (e.g., smoking, healthy eating, mood, sex), and find additional health resources based off your location or services needed.

Please note that using the app to track your medications, read articles, complete quizzes and assessments, track behaviors and find additional health resources is completely optional and is not required for participation in this study. It is solely available as a resource.

Risks and Discomforts

As with all research, there is a risk for breach of confidentiality. We try to minimize this risk by keeping your information private and secured, available to those involved with this study trained to keep information confidential, assigning a unique code to your study records instead of using your name or other identifiers on the research data we collect, and password-protecting any electronic research data.

You will be assigned to a group by chance, which may prove to be less effective or to have more side effects than the other study group or alternatives.

Benefits

You may not benefit directly from taking part in this study.

Alternatives

You can choose not to participate in the study, and this will not affect your medical care or opportunities for care coordination in any way.

Confidentiality

Information obtained about you for this study will be kept confidential to the extent allowed by law. However, research information that identifies you may be shared with people or organizations for quality assurance or data analysis, or with those responsible for ensuring compliance with laws and regulations related to research. They include:

- the UAB Institutional Review Board (IRB). An IRB is a group that reviews the study to protect the rights and welfare of research participants.
- National Institute of Mental Health (NIMH)
- the Office for Human Research Protections (OHRP)

The information from the research may be published for scientific purposes; however, your identity will not be given out in those publications.

This research is covered by a Certificate of Confidentiality from the National Institutes of Health. The researchers with this Certificate may not disclose or use information, documents, or biospecimens that may identify you in any federal, state, or local civil, criminal, administrative, legislative, or other action, suit, or proceeding, or be used as evidence, for example, if there is a court subpoena, unless you have consented for this use. Information, documents, or biospecimens protected by this Certificate cannot be disclosed to anyone else who is not connected with the research except, if there is a federal, state, or local law that requires disclosure (such as to report child abuse or communicable diseases but not for federal, state, or local civil, criminal, administrative, legislative, or other proceedings, see below); if you have consented to the disclosure,

including for your medical treatment; or if it is used for other scientific research, as allowed by federal regulations protecting research subjects.

The Certificate cannot be used to refuse a request for information from personnel of the United States federal or state government agency sponsoring the project that is needed for auditing or program evaluation by the *NIMH* which is funding this project or for information that must be disclosed in order to meet the requirements of the federal Food and Drug Administration (FDA). You should understand that a Certificate of Confidentiality does not prevent you from voluntarily releasing information about yourself or your involvement in this research. If you want your research information released to an insurer, medical care provider, or any other person not connected with the research, you must provide consent to allow the researchers to release it.

The Certificate of Confidentiality will not be used to prevent disclosure as required by federal, state, or local law of such as child abuse and neglect, or harm to self or others.

The Certificate of Confidentiality will not be used to prevent disclosure for any purpose you have consented to in this informed consent document.

As part of this study, you will be tested for HIV disease. If the results show that you are positive for *HIV*, the study staff will tell you the results. The study staff will be required to give your name to the Alabama Department of Public Health if you test positive because this is the law.

Voluntary Participation and Withdrawal

Whether or not you take part in this study is your choice. There will be no penalty if you decide not to be in the study. If you decide not to be in the study, you will not lose any benefits you are otherwise owed. You are free to withdraw from this research study at any time. Your choice to leave the study will not affect your relationship with this institution. Contact the study doctor or staff if you want to withdraw from the study.

You may be removed from the study without your consent if the sponsor ends the study, if the study doctor decides it is not in the best interest of your health, or if you are not following the study rules.

Cost of Participation

There will be no cost to you for taking part in this study.

Payment for Participation

You will be paid $50 for each survey completed and $50 for the interview. The total amount you will receive for participation is $200. Ask the study staff about the method of payment that will be used for this study (e.g., check, cash, gift card, direct deposit).

You are responsible for paying any state, federal, Social Security or other taxes on the payments you receive. You will receive a form 1099 in January of the year following your participation in this study. This form is also sent to the IRS to report any money paid to you. No taxes are kept from your payment.

Optional Research

Our research group (RISC- Research and Informatics Service Center) conducts many research studies. We would like the opportunity to contact you for future studies you may be eligible to participate in. You may change your mind at any time.

Initial your choice below:

_____ I agree to be contacted for future research studies in RISC.

_____ I do NOT agree to be contacted for future research studies in RISC

New Findings

You will be told by the study doctor or the study staff if new information becomes available that might affect your choice to stay in the study.

Questions

If you have any questions, concerns, or complaints about the research, please contact the study doctor. You may contact Dr. Latesha Elopre at 205-975-2457.

If you have questions about your rights as a research participant, or concerns or complaints about the research, you may contact the UAB Office of the IRB (OIRB) at (205) 934-3789 or toll free at 1-855- 860-3789. Regular hours for the OIRB are 8:00 a.m. to 5:00 p.m. CT, Monday through Friday.

Legal Rights

You are not waiving any of your legal rights by signing this consent form.

Signatures

Your signature below indicates that you have read (or been read) the information provided above and agree to participate in this study. You will receive a copy of this signed consent form.

____

Signature of Participant Date

____

Signature of Person Obtaining Consent Date
